# Weather and food availability additively affect reproductive output in an expanding raptor population

**DOI:** 10.1007/s00442-021-05076-6

**Published:** 2021-11-19

**Authors:** Melanie Nägeli, Patrick Scherler, Stephanie Witczak, Benedetta Catitti, Adrian Aebischer, Valentijn van Bergen, Urs Kormann, Martin U. Grüebler

**Affiliations:** 1 Swiss Ornithological Institute, Seerose 1, 6204 Sempach, Switzerland; 2 Department of Evolutionary Biology and Environmental Studies, University of Zurich, Wintherthurerstrasse 190, 8057 Zurich, Switzerland; 3 Impasse du Castel 20, 1700 Fribourg, Switzerland

**Keywords:** Food supplementation, *Milvus milvus*, Nest survival, Nestling survival, Nestling development

## Abstract

The joint effects of interacting environmental factors on key demographic parameters can exacerbate or mitigate the separate factors’ effects on population dynamics. Given ongoing changes in climate and land use, assessing interactions between weather and food availability on reproductive performance is crucial to understand and forecast population dynamics. By conducting a feeding experiment in 4 years with different weather conditions, we were able to disentangle the effects of weather, food availability and their interactions on reproductive parameters in an expanding population of the red kite (*Milvus milvus*), a conservation-relevant raptor known to be supported by anthropogenic feeding. Brood loss occurred mainly during the incubation phase, and was associated with rainfall and low food availability. In contrast, brood loss during the nestling phase occurred mostly due to low temperatures. Survival of last-hatched nestlings and nestling development was enhanced by food supplementation and reduced by adverse weather conditions. However, we found no support for interactive effects of weather and food availability, suggesting that these factors affect reproduction of red kites additively. The results not only suggest that food-weather interactions are prevented by parental life-history trade-offs, but that food availability and weather conditions are crucial separate determinants of reproductive output, and thus population productivity. Overall, our results suggest that the observed increase in spring temperatures and enhanced anthropogenic food resources have contributed to the elevational expansion and the growth of the study population during the last decades.

**Supplementary Information:**

The online version contains supplementary material available at 10.1007/s00442-021-05076-6.

## Introduction

Identifying the drivers of reproductive output and quantifying the associated variation in individual fitness is crucial for understanding and forecasting species’ population dynamics (Lindström 1999; Newton and Brockie 2003). In the light of current climate change, the effects of weather conditions on reproductive output become critically important due to their demographic consequences (Møller et al. 2010), particularly in long-lived species (Vedder et al. 2013). Adverse weather conditions such as heat waves (Hansen 2009; Conradie et al. 2019) or heavy rainfalls (Kalcounis-Rueppell et al. 2002; Cayuela et al. 2016; Linton et al. 2018; Plard et al. 2019) restrict reproductive output in many vertebrates. Moreover, weather effects on early development of juveniles can carry over to affect survival and reproduction during later life stages, thereby shaping population dynamics (e.g., Descamps et al. 2008). However, weather conditions can interact with other environmental factors in complex, yet little understood ways, because adverse weather can influence the breeding environment through multiple paths (Kleijn et al. 2010; Hallinger and Cristol 2011; Arbeiter et al. 2016).

Food availability represents a key driver of reproductive output and is often affected by altered land use and other anthropogenic activities (Newton and Brockie 2003; Fuller 2012). It is well known for many species that low food availability can reduce the number (Rode et al. 2006; Tamburi and Martín 2011), survival (Wauters and Lens 1995; Perrig et al. 2014), and body condition of offspring (Therrien et al. 2008). However, food conditions often strongly correlate with weather conditions, because adverse weather can not only affect the brood, but the availability and accessibility of food, as well as foraging costs of parents (Grüebler et al. 2008; Schifferli et al. 2014). Thus, to understand weather effects on reproductive performance, it is crucial to disentangle direct effects on the brood (e.g., effects on nestling thermoregulation) from the indirect food-mediated effects (Steenhof et al. 1997; Dawson and Bortolotti 2000). To this end, an experimental approach is called for. Moreover, increasing evidence for interacting effects of environmental drivers on animal reproduction (Steenhof et al. 1997; Scopel and Diamond 2018) suggests that weather and food interact to affect reproduction; improved food availability may dampen the detrimental effect of inclement weather on nestling survival (Fisher et al. 2015). This is particularly expected in long-lived species, where increased foraging costs due to low food availability reduce brood survival, rather than parent survival (Promislow and Harvey 1990; Jönsson 1997). Yet experimental evidence for such interactions remain scarce in birds, because they require replicates across large spatial or temporal scales to cover sufficient variation in weather conditions (but see Fisher et al. 2015).

In birds, the effects of adverse weather and low food availability on reproductive output might vary over the course of a breeding attempt. This is because the reproductive investment of the parents into the brood and offspring susceptibility to weather conditions can change with increasing brood age (Ghalambor and Martin 2000; Zwaan et al. 2020). This often translates to elevated rates of brood loss during the early phase of the breeding attempt (Grant et al. 2005; Wilson et al. 2007). During the incubation phase, adverse weather conditions mainly affect incubation behaviour, often leading to longer recess times (MacDonald et al. 2013; Coe et al. 2015). In contrast, during the nestling phase, weather conditions affect both parental care and nestling physiology (Anctil et al. 2014; Öberg et al. 2015; Ouyang et al. 2015). Nestling survival and development are shown to be negatively affected by rain and cold temperatures, particularly in the first days after hatching (Jovani and Tella 2004) when thermoregulation is not yet fully developed (Whittow and Tazawa 1991). Thus, a counteracting effect of high food availability under inclement weather conditions may differ between the incubation and the nestling phase.

Food supplementation experiments are often applied to quantify the effect of food availability on reproductive behaviour and performance (Dewey and Kennedy 2001; Dawson and Bortolotti 2002; Ruffino et al. 2014). Many studies showed that food supplementation increases the survival of nestlings, alters adult behaviour and brood sex ratio (Robb et al. 2008). Yet, only few experiments aimed at disentangling the influence of food from other environmental effects. It is well established that food supplementation interacts with natural food availability, such that the effect of supplementation on reproductive output is stronger when natural food availability is low than when it is high (Byholm and Kekkonen 2008; Grüebler et al. 2018). In contrast, food supplementation experiments to investigate food-weather interactions are rare, likely because it remains a logistical challenge to simultaneously perform food supplementation treatments and capture the required variation in weather conditions in studies that are typically limited in their spatial or temporal extent.

In this 4-year experimental study, we investigate the effects of weather and food availability on multiple reproductive parameters in an expanding population of a long-lived, conservation-relevant raptor species, the red kite (*Milvus milvus*). More specifically, we assess whether experimentally enhanced food availability can mitigate the negative effects of adverse weather on reproductive performance during the incubation and the nestling phase, i.e., whether interacting or additive effects occur. We expected that adverse weather conditions and low natural food availability reduce reproductive performance, while food supplementation enhances it, but that the importance of these effects differs between reproductive phases and siblings of different age (see Morandini and Ferrer 2015). We expected that the negative effect of weather conditions on reproductive performance is reduced in food-supplemented broods compared to unsupplemented control broods, and that this effect also differs between reproductive phases. The results of this study provide deeper insights into the interplay between the effects of adverse weather conditions and food availability on reproductive performance, and thus, into how recent changes in weather and food conditions may have contributed to the observed population increase of the red kite in Switzerland.

## Methods

### Study area and study species

The study area is located in western Switzerland in the cantons of Freiburg and Bern, and has an extent of approximately 387 km^2^. It ranges from the lowlands of the Swiss plateau to a more mountainous area towards the Swiss Alps (482–1763 m.a.s.l), and is characterized by agriculture (56.25%), managed forests (26.95%), settlements (8.4%) and unproductive land (8.4%). The agriculture is dominated by dairy farming and meat production, resulting in large meadow areas, and thus, potential red kite breeding habitat (StatA 2018). The red kite is a large near-endemic European raptor species and is a priority species for conservation in Switzerland. While populations in the main distribution area of central and southern and Europe (Germany, France and Spain) partially still suffer from considerable decreases, in Switzerland, the species showed a rapid recovery from under 100 breeding pairs in the 1950s to more than 3000 breeding pairs in 2018 (Knaus et al. 2018). During the last decades, the population in the study area has increased from zero to a high density of up to 40 pairs per 100 km^2^. The red kite breeds in trees and raises 1–4 nestlings per breeding season (Aebischer 2009). Incubation usually lasts between 31 and 35 days. After hatching, nestlings stay in the nest for about 40–50 days (age including incubation 71–85 days), but only start to fly at c. 50–55 days of age (age including incubation 81–90 days) (Aebischer 2009). Both parents contribute to the rearing of the nestlings, while the female carries out a larger part of the incubation than the male. It is a facultative scavenger species that regularly visits anthropogenic feeding sites (for example agricultural compost heaps and feeding by private residents), in the study area (Cereghetti et al. 2019) and tends to monopolize them (Welti et al. 2019).

### Focal nests and nestling parameters

In 2015–2018, we monitored possible red kite territories from March to July. When an active nest was found, we observed it every 7–14 days using a scope to assess the start of incubation. In total we monitored 418 nests containing 559 nestlings (Table 1). At some nests, we additionally installed cameras (*N* = 143) either before incubation or during the nestling phase (when the nestlings were old enough to maintain proper thermoregulation) to closely monitor behaviour at the nest. Cameras were placed at least 2 m away from the nest or on a neighbouring tree whenever possible to minimize disturbance. After incubation started, we reduced observation effort until shortly before the assumed hatching date, when the frequency of observations was increased to estimate hatching date. Nests were climbed and nestlings measured for the first time when they were 15–22 days. After the first measurements, survival of nestlings was checked during one to four additional measuring events, by camera or by observation with scope until shortly before fledging (nestling age = 35–45 days) when we took the last measurements not to risk premature fledging. We recorded body mass, primary feather lengths (P8) and wing length of the nestlings at every measurement. Blood samples were taken for genetic sex determination (*N* = 429). DNA was extracted and purified using the QIAGEN DNeasy Blood and Tissue Kit and afterwards analysed with PCR amplification of the CDH1 gene in the avian sex chromosome, using primers 2550 and 2718 (Fridolfsson and Ellegren 1999).

**Table 1:** Sample sizes in experimental groups, separated by model

	Control			Food supplementation		
Model	Nestlings	Nests	Measurements	Nestlings	Nests	Measurements
*Total Monitored*	*–*	*321*	*–*	*–*	*97*	*–*
Nest Survival	–	261	–	–	83	–
*Total Measured*	*440*	*239*	*829*	*119*	*66*	*321*
Nestling Survival	289	129	–	109	54	–
Brood Size at Fledging	–	237	–	–	65	–
Body Mass	376	214	723	103	60	269

Total sample sizes are in italics and model-specific sample sizes in normal letters

The line separates between sample sizes of data including incubation, and sample sizes of data from nestling phase only

Previous studies recommend primary feather length as a useful measure to estimate nestling age in red kites (Traue and Wuttky 1966; Mougeot et al. 2011; Pfeiffer and Meyburg 2015). Accordingly, we aged all nestlings with unknown hatching date using a growth curve of the eighth primary feather (see Electronic Supplementary Material S1). We calculated incubation start by subtracting the average incubation length (mean = 31.6 ± 1.2 (SD)) from the hatching date (calculated by feather length) of the brood, or by estimation based on the nest observations (incubation start or hatching date) for nests without nestling measurements. Furthermore, we generated two binary variables for hatching order (first-hatched and last-hatched) based on the hatching dates of the nestlings within a nest. Singletons were considered as first-hatched nestlings.

### Supplementary feeding experiment

To experimentally quantify the effect of food availability on reproductive performance, we manipulated food resources by offering dead day-old chickens to the breeding birds (2015: *N* = 10 pairs, 2016: *N* = 29, 2017: *N *= 37, 2018: *N* = 12). We placed five chickens (mean weight per chick = 38 ± 2.3 g (SD)) per adult and per nestling younger than 10 days of age, and ten chickens per nestling older than 10 days every other day on wooden platforms located 20–200 m from the target nests until the nestlings were fledged (Baucks 2018). The amount of food provided exceeded previously reported daily energy requirements of 150 g per nestling (Wasmund 2013). We assessed whether the food supplementation was accepted by observing the nest shortly after a feeding event. We included nests, where the food supplementation was not accepted in the supplemented group to avoid self-selection bias (2015: *N *= 2 nests, 2016: *N* = 17, 2017: *N* = 4, 2018: *N *= 1), hereby yielding a conservative estimate of the feeding effect. We acknowledge that our study does not account for anthropogenic food sources that occurred outside of our experimental treatment, and which are widespread in the study area (Cereghetti et al. 2019). Given that anthropogenic feeding sites might reduce the relative effect of experimental feeding, our results should be considered as conservative estimates of the experimental feeding effects.

### Environmental factors

To estimate the natural food availability, we monitored the rodent activity in a total of 180 monthly transects representing the main agricultural habitat types in the region, evenly distributed across four sub-regions (following Apolloni et al. 2018), and derived a monthly rodent activity index for the study region (see Electronic Supplementary Material S2). To characterize the weather, we used data from the MeteoSchweiz weather station located within the study area in Posieux-Freiburg: mean total precipitation (from 06:00 to 18:00, in mm, denoted as: rain), mean wind speed (whole day, in km h^−1^, denoted as: wind), and mean temperature (whole day, in °C, denoted as: temperature).

### Statistical analyses

Statistical analyses were conducted in R (R version 3.6.3, R Core Team 2018). We investigated the effects of year, food supplementation, rodent activity, rain, temperature, wind, and additional model specific control variables on nest survival, nestling survival, number of fledglings and fledgling body mass. As such, we ran four sets of analyses. For all analyses, we checked for correlation between explanatory variables. When a Pearson correlation or Kruskal–Wallis coefficient of *r *≥ 0.7 was found, one of the correlated variables was excluded (Tabachnick and Fidell 2013) (only one case when we decided to exclude wind due to the correlation with rodent activity). In three models (analyses of nestling survival, number of fledglings, and body mass), year showed an *r* ≥ 0.7 with at least one of the other explanatory variables. Thus, we applied a two-step approach for all four models: first, we ran a model for every response variable including year (year model) instead of environmental variables, which enabled quantifying annual differences. Second, we ran these models with all environmental factors without year (environmental model), to investigate the potential environmental causes for the observed annual differences. Food supplementation treatment was included as a categorical variable into both models. Furthermore, rodent activity was moderately correlated with wind (*r* = 0.58), and both variables were thus included individually as well as together for the model selection. The initial models included the following ecologically meaningful interactions between uncorrelated (*r* < 0.5) explanatory variables: year × food supplementation, food supplementation × environmental factors, and weather variables × rodent activity. Further model-specific twofold and threefold interactions are described in the corresponding sections, below. Sample sizes for each model are given in Table 1.

#### Nest survival analysis

Daily nest survival rates were estimated with the package RMARK (Laake 2013). We separated the breeding period into two phases, the incubation phase and the nestling phase. The incubation phase included also the first 3 days of the nestling phase (*d* = 35), as nestlings younger than 4 days cannot be seen with a scope. The nestling phase lasted from brood age 36 to brood age 72 (age of nestlings 4–45 days), shortly before fledging. Reproductive phase, year, and the environmental factors (average of the daily mean values over the brood-specific period of the phase) were included into the analysis as unstandardized individual covariates. Additional twofold interactions between phase, year, food supplementation, and environmental factors were included into the initial model. A three-way interaction between year, phase, and food supplementation was considered, but had to be excluded due to a too small sample size. Furthermore, we tested the following three-way interactions to assess how environmental factors influenced the two phases: rain, phase, and food supplementation; temperature, phase, and food supplementation; rodent activity, phase, and food supplementation; wind, phase, and food supplementation. To evaluate which candidate model fits the data best, we used the built-in AICc values of the MARK program (Burnham and Anderson 2004; Dinsmore and Dinsmore 2007). Phase-specific and total nest survival estimates were calculated by multiplying daily survival rates (DSR). The respective variances were calculated using the delta method (Powell 2007).

#### Analyses of nestling survival, number of fledglings, and body mass

In these analyses, we applied linear mixed-effect models using the package lme4 (Bates et al. 2015). We used a full-model approach, keeping all main effects in the model, but excluding insignificant interactions. We standardized continuous variables by subtracting the mean and dividing by the standard deviation. As a measure of parameter uncertainty, we estimated 95% Bayesian credible intervals (CrI) (Korner-Nievergelt et al. 2015). We checked for normal distribution (package ‘arm’ by Gelman and Su 2018) and temporal autocorrelation of residuals (with the acf function). In the analyses of nestling survival and body mass, we first implemented brood ID nested in nest ID as a random effect. However, as only few nests were used multiple times by the pairs, the models were over-fitted, and we only included brood ID in the nestling survival analysis, nest ID in the number of fledglings analysis, and brood ID and bird ID in the body mass analysis.

We used a generalized linear mixed model with binomial distribution and logit link to estimate survival rates of nestlings between the first and the last observation. We included only broods with at least two nest visits and ≥ 1 surviving nestling to consider mechanisms affecting survival of individual nestlings rather than survival of the entire brood (for nest survival see above). As the age at the first visit and the time between visits was highly correlated (*r* = 0.84), we controlled for age at first visit in the analysis, but not for the time between visits. In addition to the standard variables, we included the two variables of hatching order and the number of nestlings as explanatory factors. We averaged the environmental variables between hatching date and last brood measurement. Because environment might affect nestlings of varying ranks differently, two-way interactions between hatching order and environmental variables (including year and supplementation treatment) were included into the initial model. To evaluate the separate effect of food supplementation during adverse weather conditions on last-hatched nestlings, we included three-way interactions between environmental variables, last-hatched and food supplementation.

In the model analysing factors affecting the number of fledglings, the response variable was brood size at the last measurement of a brood, independent of age. Therefore, brood age at the last measurement (44–87 days) was added as a control variable to the same set of explanatory variables used in the analysis of nestling survival. For the analysis of body mass, we conducted an orthogonal polynomial regression, including the length of the eighth primary feather, and its second polynomial as explanatory variables to control for the age-dependence of body mass. The environmental factors entered the model as average values over the week before the measurement, and the rodent activity was used from the month when the measurement was taken. We also included sex, hatching order and number of nestlings as explanatory variables. To control for differences in growth rates, we added the two-way interactions between feather length and sex, hatching order, brood size and environmental variables (including year and supplementary treatment) and the three-way interaction between last-hatched, feather length and food supplementation to the variable set of the analysis of nestling survival.

## Results

### Nest survival

Between 2015 and 2018 we monitored a total of 418 red kite nests (Table 1), of which 61 could be visited only once; these nests were excluded from further analysis. To exclude the possibility of an experimentation bias, we excluded thirteen additional nests from further analysis, where nest failure occurred within 48 h after the climbing event. This led to a total sample size of 344 nests (Table 1).

**Fig. 1:**
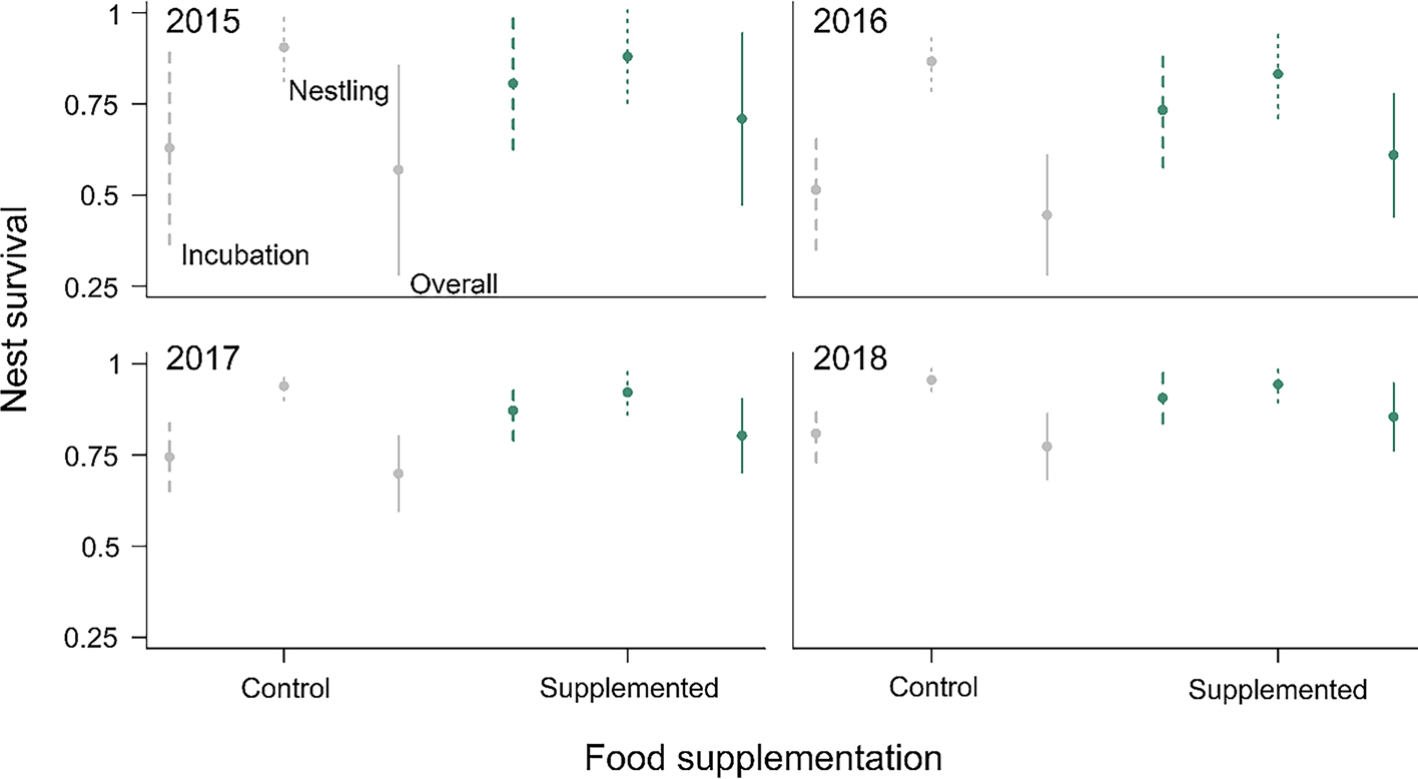
Predicted values of nest survival rates of control (grey) and food supplemented (green) red kite nests in the different years for the incubation phase (dashed line), the entire breeding season (solid line) and the nestling phase (dotted line), separately. Mean nest survival and 95% confidence intervals of the year model with the lowest AICc are shown

**Fig. 2:**
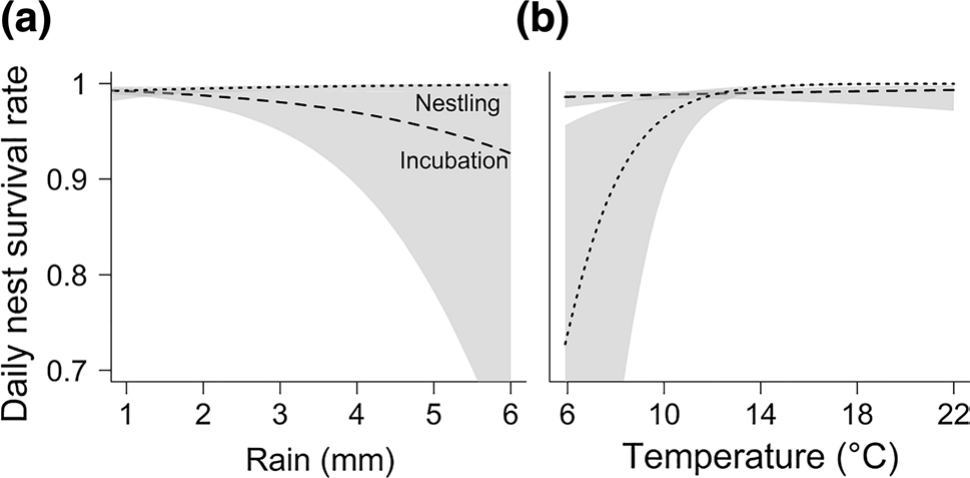
Predicted values of daily nest survival rates of red kites for the incubation phase (dashed line) and the nestling phase (dotted line) of the single best MARK environmental model, **a** in relation to the mean daily amount of rain during the phase, and **b** in relation to mean daily temperature during the phase. Shaded area represents 95% confidence interval

The data did not support a single best year model explaining nest survival, as three models had similar AICc values ranging within ΔAICc < 2 (Table 2a). All three models indicated that nest survival differed among years, and nest survival was lower during incubation than during the nestling phase (unsupplemented control nests: incubation phase mean = 67.40% ± 5.89% (CI), nestling phase = 91.62% ± 2.67%; Fig. 1). Models also revealed high support for an effect of food supplementation on nest survival (all high-ranked models except the third ranked model). The top ranked model was the only year model supporting an interaction between phase and food supplementation. However, model selection of environmental models showed clear support for phase-specific effects of food supplementation. The single best environmental model included the interaction between phase and food supplementation, providing support for phase-specific effects of food supplementation (Table 2b). Food-supplemented nests had higher nest survival during the incubation phase compared to control nests (nest survival: supplemented nests = 84.30% ± 5.46% (CI), control = 70.34% ± 6.43%), whereas we found no effect of food supplementation on nest survival in the nestling phase (nest survival: supplemented nests = 67.24% ± 12.06% (CI), control = 80.00% ± 6.07%). In addition, the top ranked model included the interactions between phase and rain, and between phase and temperature, suggesting that annual variation in nest survival was caused by a negative effect of rain during the incubation phase, and low temperatures during the nestling phase (Fig. 2). Finally, we found no support for an effect of rodent activity or wind on nest survival (ΔAICc > 5.72).

**Table 2:** Model selection results for the MARK nest survival analysis (*N* = 344 nests) for (a) year models and (b) environmental models

(a)	Year models			
Model	AICc	ΔAICc	weight	deviance
Food suppl. + year + phase + food suppl. × phase	635.08	0.00	0.37	621.07
Food suppl. + year + phase	635.94	0.86	0.24	623.93
Year + phase	636.66	1.58	0.17	626.66
Food suppl. + year + phase + food suppl. × phase + year × phase	637.47	2.39	0.11	617.46

(b)	Environmental models			
Model	AICc	ΔAICc	weight	deviance
Food suppl. + temperature + rain + phase + phase × temperature + food suppl. × phase + phase × rain	625.32	0.00	0.47	609.31
Temperature + rain + phase + phase × temperature + phase × rain	627.38	2.07	0.17	615.38

Only the top models with ΔAICc < 2.5 are shown

### Nestling survival

Of the 192 nests that were measured multiple times, 183 had at least one surviving nestling, leading to a sample size of 398 nestlings (Table 1) of which 47 (11.8%) died before the last measurement. Average nestling age at the first visit was 20 ± 9 days (SD), and at the last visit, 41 ± 5 days. Mean difference between first and last visit was 21 ± 9 days.

**Fig. 3:**
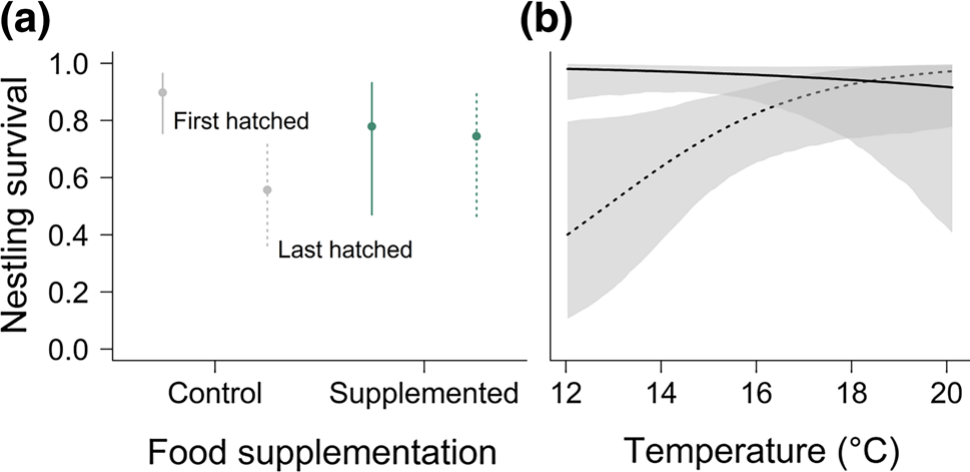
Predicted values of nestling survival for first-hatched (solid line) and last-hatched (dashed line) red kite nestlings in **a** food-supplemented and control broods, and **b** at different mean daily temperatures. Results of the environmental model are shown. Error bars and shaded areas represent 95% credible intervals. Age at first measurement was set to day 10

We found a significant interaction between food supplementation and last-hatched nestling in both the year and the environmental model (Table 3a), indicating that food supplementation increased survival in last-hatched, but not in earlier-hatched nestlings. The prediction for 10-day-old last-hatched nestlings revealed a 19%-increase in survival probability in supplemented compared to unsupplemented nestlings (Fig. 3a). In the environmental model, low temperatures considerably reduced the survival of last-hatched, but not that of earlier-hatched nestlings (Fig. 3b). The binned residual plots indicated that nestling survival was generally overestimated for low survival rates.

**Table 3:** Model estimates for (a) the nestling survival model (*N* = 398 nestlings), (b) the brood size at fledging model (*N* = 302 broods), and (c) the body mass model (*N* = 992 measurements)

(a)	Nestling Survival					
	Year model			Environmental model		
Explanatory variable	Estimate	95% CrI		Estimate	95% CrI	
Intercept	4.89	1.59	8.61*	5.86	2.78	8.74*
Food supplementation	– 0.99	– 2.31	0.43	– 0.93	– 2.42	0.49
Brood size 2	– 1.20	– 3.98	1.44	– 1.22	– 3.91	1.55
Brood size 3	– 2.39	– 5.15	0.32	– 2.29	– 4.90	0.50
First-hatched	– 0.58	– 1.98	0.90	– 0.68	– 2.12	0.77
Last-hatched	– 2.50	– 3.80	– 1.09*	– 2.58	– 4.02	– 1.23*
Age	0.60	0.16	1.04*	0.65	0.19	1.13*
2016	– 0.07	– 2.18	1.89	–	–	–
2017	1.59	– 0.30	3.45	–	–	–
2018	0.59	– 1.36	2.46	–	–	–
Wind	–	–	–	– 0.44	– 0.99	0.10
Temperature	–	–	–	– 0.32	– 1.14	0.48
Rain	–	–	–	– 0.03	– 0.62	0.56
Rodent activity	–	–	–	– 0.60	– 1.26	0.02
Food suppl. × last-hatched	1.83	0.12	3.55*	1.72	0.07	3.53*
Temperature × last-hatched	–	–	–	1.13	0.27	2.00*
Random factor		SD			SD	
Brood ID		1.06			1.08	

(b)	Brood size					
	Year model			Environmental model		
Explanatory variable	Estimate	95% CrI		Estimate	95% CrI	
Intercept	1.48	1.17	1.76*	1.79	1.62	1.96*
Food supplementation	0.03	– 0.17	0.22	– 0.03	– 0.22	0.17
2016	0.06	– 0.24	0.37	–	–	–
2017	0.42	0.14	0.70*	–	–	–
2018	0.35	0.07	0.64*	–	–	–
Age	0.00	– 0.08	0.08	0.03	– 0.06	0.12
Temperature	–	–	–	– 0.11	– 0.22	– 0.01*
Rain	–	–	–	– 0.13	– 0.23	– 0.04*
Rodent activity	–	–	–	0.09	0.00	0.18*
Random factor		SD			SD	
Nest ID		0.17			0.17	

(c)	Body mass (g)					
	Year model				Environmental model	
Explanatory variable	Estimate	95% CrI		Estimate	95% CrI	
Intercept	806.4	759.1	854.5*	874.8	851.5	898.4*
Feather	207.9	157.4	257.5*	153.1	145.9	160.8*
(Feather)^2^	– 42.9	– 48.2	– 37.6*	– 41.8	– 47.0	– 36.6*
Food supplementation	30.5	8.5	51.9*	21.3	– 1.6	42.8
Brood size 2	0.7	– 19.5	21.9	– 1.4	– 20.8	19.4
Brood size 3	– 4.9	– 31.8	21.0	– 1.5	– 26.3	24.2
First-hatched	0.5	– 16.2	17.4	0.2	– 16.6	17.0
Last-hatched	– 38.7	– 55.9	– 21.2*	– 37.7	– 54.5	– 19.9*
Male nestling	– 64.7	– 76.3	– 52.9*	– 64.8	– 76.3	– 53.0*
2016	43.8	– 3.2	91.6	–	–	–
2017	67.9	20.5	113.3*	–	–	–
2018	82.8	36.0	128.2*	–	–	–
Wind	–	–	–	– 11.6	– 17.7	– 5.9*
Temperature	–	–	–	– 9.9	– 17.6	– 2.1*
Rain	–	–	–	– 5.9	– 11.5	– 0.0*
Rodent activity	–	–	–	28.6	14.6	42.8*
Food suppl. × feather	– 6.9	– 16.4	2.8	– 6.9	– 16.5	2.5
Food suppl. × (Feather)^2^	– 17.5	– 27.2	– 8.2*	– 17.1	– 26.6	– 7.6*
Male nestling × feather	– 13.7	– 21.6	– 5.5*	– 11.9	– 20.8	– 3.7*
Feather × 2016	– 42.5	– 95.0	9.9	–	–	–
Feather × 2017	– 62.5	– 113.0	– 11.4*	–	–	–
Feather × 2018	– 60.7	– 110.9	– 8.9*	–	–	–
Food suppl. × rodent activity	–	–	–	– 26.3	– 44.8	– 7.9*
Brood size 2 × rodent activity	–	–	–	– 17.2	– 33.7	– 1.2*
Brood size 3 × rodent activity	–	–	–	– 28.3	– 47.6	– 9.9*
Random factor		SD			SD	
Brood ID		61.5			60.4	
Bird ID		20.6			21.1	

Results for two alternative models, the year model and the environmental model are shown

*95% CrI* = 95% Credible intervals

Significance for explanatory variables is indicated by an asterisk

^2^ indicating that we included the squared feather length in the model

### Number of fledglings

Three nests were not available for the analysis due to missing brood age resulting in a sample size of 302 nests (Table 1). Broods had, on average, 1.77 ± 0.7 fledglings (SD) across years, and the number of fledglings was counted at an average age of 38 ± 7.4 days (SD). In the environmental model, wind had to be excluded due to a high correlation with rodent activity. In both models, food supplementation showed neither a significant main effect, nor a significant interaction effect on the number of fledglings. Successful broods showed significantly more fledglings in 2017 (mean = 1.89, CrI = 1.71, 2.09) and 2018 (1.83, CrI = 1.60, 2.05) than in 2015 (1.48, CrI = 1.19, 1.76). The environmental model indicated that the annual differences were related to rodent activity, our index of natural food availability. Rodent activity increased from 2016 to 2018 and had a significant positive effect on the number of fledglings, whereas high temperature and average rain since hatching were associated with a reduced number of fledglings (Table 3b).

### Body mass

**Fig. 4:**
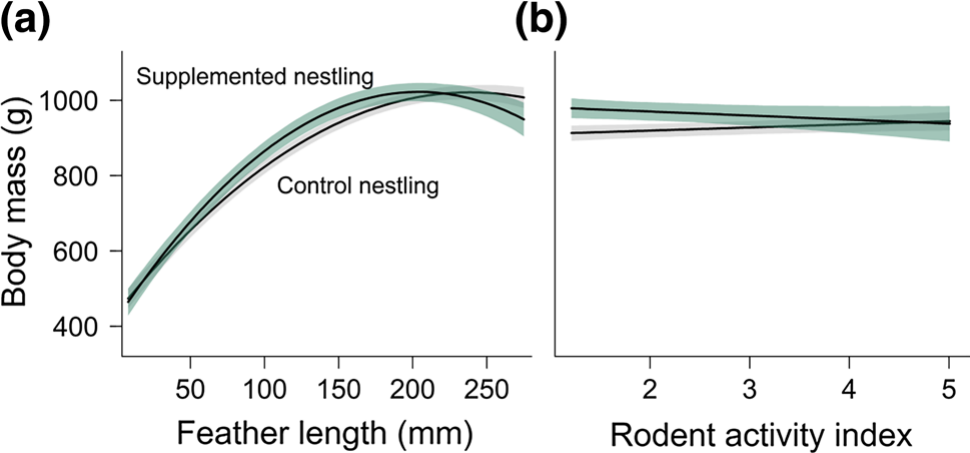
Predicted values of body mass of food-supplemented and control red kite nestlings **a** in relation to the eighth primary feather length, and **b** in relation to the rodent activity index. Feather length was set to the average (141 mm). The results of the environmental model are shown. Shaded areas represent 95% credible intervals

Two nestlings were found dead on the ground when the nests were visited for measuring, and thus, were excluded from the analysis. We had to exclude a further 138 individuals, either because of missing sex (*N* = 80), feather length (*N* = 44), hatching order (*N* = 7), or body mass (*N* = 7). This led to a total of 992 measurements of 479 nestlings that entered the analysis (see Table 1). Both models revealed differences in growth patterns between nestlings of different rank and sex. We found lower body mass for last-hatched and male nestlings (Table 3c). The model supported a significant interaction between feather length and sex, indicating that the weight difference between the heavier females and the males was amplified with advanced nestling age (Δ_mass_ male vs. female: feather length 20 mm = 40.48 g; feather length 250 mm: Δ_mass_ = 85.86 g). A significant interaction between food supplementation and the second polynomial of feather length indicated that food-supplemented and control nestlings differed in their growth pattern as a non-linear function of feather growth. While both experimental groups showed a similar weight at young age (short feathers), food-supplemented nestlings were about 51.44 g heavier than control nestlings at intermediate age (i.e., feather length = 140 mm; Fig. 4a). This difference disappeared again in old nestlings (feather length = 250 mm, Fig. 4a), indicating that food-supplemented nestlings reached fledging weight earlier. During the linear growth phase, nestlings were lighter in the years with low (2015 and 2016) compared to years with high (2017 and 2018) rodent activity. These annual differences in weight disappeared by the time of fledging (Table 3c). The environmental model indicated that food supplementation increased body mass when rodents were scarce, but the effect disappeared when rodent activity was high (Fig. 4b). The interaction between brood size and rodent activity was significant, suggesting that broods with one nestling benefited more from high rodent availability than broods with two or three nestlings (Table 3c). Temperature, rain and wind negatively affected body mass, with a 5.91%, 9.87% and 11.58% decrease along the corresponding gradients, respectively.

## Discussion

The results of our experimental study show that both weather conditions and food availability additively affect reproductive performance in the red kite. Rain and low food availability were the strongest drivers of brood loss during incubation, whereas low temperature was the dominant driver during the nestling phase. Adverse weather and low rodent densities reduced the number of fledglings, reducing survival of the last-hatched nestling in particular. The positive effect of experimental food supplementation on reproductive output was phase-specific and mediated through the nestlings’ environment: food supplementation increased nest survival during incubation, and increased survival in last-hatched, but not in early hatched nestlings. Furthermore, food supplementation increased body mass, particularly under low natural food availability, potentially carrying-over to later life-history stages (Fattebert et al. 2019). These results suggest that changes in climate and food availability during the breeding season might be one of the important drivers of the recent population increase in red kites in Switzerland.

### Additive versus interactive effects

Avian studies showed that foraging success (Sergio 2003), incubation time (MacDonald et al. 2013), food provisioning (Dawson and Bortolotti 2000), and parental foraging effort (Schifferli et al. 2014) are often reduced during periods of adverse weather, suggesting higher costs for parents during these periods (Edward and Chapman 2012). Food supplementation is, therefore, expected to reduce the negative effects of adverse weather on parental care behaviour, and thus, on reproductive performance (Fisher et al. 2015). Yet, we did not find that food supplementation dampened the negative effects of adverse weather on reproductive performance, despite evidence that adverse weather conditions affect parental foraging behaviour and feeding rates in our study system (Baucks 2018; Andereggen 2020). This might be explained by three potential mechanisms.

First, if the direct negative effects of adverse weather on eggs or nestlings can be diminished neither by increased nest attendance nor by increased food supply, then parents may not adjust their behaviour when supplemented food is provided under adverse weather conditions. However, this explanation seems unlikely, because females adjust nest attendance in response to rain and low temperatures, and food supplementation changed these parental adjustments during adverse weather conditions (Andereggen 2020). Second, parental use of supplemented food may be strongly reduced during adverse weather conditions, for example if inclement weather increases competition for the supplemented food by increasing platform use by non-target birds, or if adverse weather increases flight costs considerably. However, since many red kites also tolerate cold weather conditions, while staying in the study area in winter, we would expect such an effect during rainy but not during cold periods of the breeding season. Third, parents might allocate the additional food to their brood only during normal weather conditions but consume it themselves during adverse weather conditions to ensure self-maintenance. This mechanism is supported by the fact that food supplementation reduced rather than increased nest attendance during adverse weather conditions (van Bergen 2019). Moreover, food supplementation did not reduce mortality of last-hatched chicks during adverse weather conditions, which would be expected if food supplementation would result in higher feeding rates. Thus, the expected interactive effect between weather conditions and food availability on current reproduction might be absent, because, under adverse weather conditions, parents shift the food allocation from the current brood to self-maintenance, with potential gains for future reproductive output. In turn, this may indicate that weather conditions possibly affect the trade-off of investing food resources into current versus future reproduction.

### Incubation versus nestling phase

Brood loss occurred more frequently during the incubation than during the nestling phase, which is also supported by other studies of raptors (e.g.,Varland and Loughin 1993; Charter et al. 2007). In a year with low rodent density, unsupplemented nests had 21.9% lower survival probability than supplemented nests. Furthermore, an increase of 2 mm rain per day during the incubation phase raised brood loss by over 10%. Parents seem to be very sensitive to environmental conditions and rapidly abandon the brood when conditions deteriorate, likely due to elevated stress levels as a consequence of the energetic trade-off between nest attendance and self-maintenance (Thierry et al. 2013). The fact that sensitivity to adverse environmental conditions is high in the incubation phase suggests that incubation is very costly for females (Monaghan 2008). Moreover, since incubating females are mainly fed by foraging males, the results point towards complex interactions between environment, female physiological state, and partner parental investment (Wiehn and Korpimäki 1997; Ouyang et al. 2015). Thus, since weather and food conditions during the incubation phase are critical determinants for the extent of brood loss, they decisively regulate the productivity of the population.

After hatching, the number of nestlings can be altered by current environmental conditions (Mock 1985, 1994; Valkama et al. 2002; González et al. 2006). Nevertheless, environmental conditions during the nestling phase influenced the final reproductive output less than during the incubation phase. Only long periods of low temperatures during the nestling phase led to brood loss, and survival of the last-hatched nestling in successful broods was slightly reduced by low temperatures and increased by food supplementation. However, nestling development and body mass at fledging was sensitive to adverse weather and food conditions, also resulting in altered nestling stress physiology (Catitti 2018). Since there is good evidence for nestling development and fledging condition affecting post-fledging survival and dispersal in birds, in general (Matthysen 2012; Naef‐Daenzer and Grüebler 2016), and in red kites, in particular (Scherler 2020), it is likely that adverse weather and food conditions during the nestling phase carry-over to later life-history stages.

### Population consequences

The red kite is a species of national conservation concern in Switzerland (Keller et al. 2010) and in the EU (EU birds directive 2009/147/EC, Annex 1). For a long time, it has been listed as globally near threatened because of a suspected population decline, but recent population increases across much of its distribution range, including Switzerland (Knaus et al. 2018), have led to a reclassification to least concern in 2020 (BirdLife International 2020).

The results of our experimental study provide novel insights into potential drivers of this observed population growth. We observed that weather conditions and food availability influenced the reproductive output. Consequently, changes in these environmental factors, e.g., by climate change, are likely to affect red kite population dynamics. Indeed, spring temperatures in Switzerland have risen over the last decades (MeteoSchweiz 2020), which may have increased reproductive output. We hypothesize that this positive temperature effect on reproduction may have been particularly accentuated at higher elevations, which coincides with the observed elevational expansion in Switzerland (Knaus et al. 2018).

Finally, raptors are commonly fed in the study region, either intentionally by providing food directly to red kites or unintentionally through disposal of organic waste. Up to 12% of households within our study area regularly feed raptors intentionally or unintentionally (Cereghetti et al. 2019), some of which feed at higher rates than we did in our food supplementation treatment. Importantly, anthropogenic feeding likely increased over the last decades (Cereghetti et al. 2019). In conclusion, our study suggests that warming spring temperatures and enhanced anthropogenic food sources contribute to the population increase of the red kite in Switzerland.

## Supplementary Information

Below is the link to the electronic supplementary material.

Supplementary file1 (DOCX 135 kb)

## Declarations

### Availability of data

Data can be accessed on https://doi.org/10.5281/zenodo.5666252.

### Code availability

Code is available on https://doi.org/10.5281/zenodo.5666252.
