# Supplementary material for: Weather and food availability additively affect reproductive output in an expanding raptor population
Source: Oecologia. 2021 Nov 19;198(1):125–38. doi: 10.1007/s00442-021-05076-6 (PMC8803806; doi:10.1007/s00442-021-05076-6)
Supplement: Supplementary file 1 — Supplementary file1 (DOCX 135 kb) [file 442_2021_5076_MOESM1_ESM.docx]

# Electronic Supplementary Material

# Weather and food availability additively affect reproductive output in an expanding raptor population

Melanie Nägeli^1,2^, Patrick Scherler^1,2,*^, Stephanie Witczak^1,2^, Benedetta Catitti^1,2^, Adrian Aebischer^3^, Valentijn van Bergen^1^, Urs Kormann^1^ & Martin U. Grüebler^1^

^1^ Swiss Ornithological Institute, Seerose 1, CH-6204 Sempach, Switzerland

^2^ Department of Evolutionary Biology and Environmental Studies, University of Zurich, Wintherthurerstrasse 190, CH-8057 Zurich, Switzerland

^3^ Impasse du Castel 20, CH-1700 Fribourg, Switzerland

*Corresponding author: [Patrick.scherler@vogelwarte.ch](mailto:Patrick.scherler@vogelwarte.ch)

# S1: Nestling age calculation

Primary feather length is a useful measure to estimate nestling age in red kites (Traue and Wuttky 1966; Mougeot et al. 2011; Pfeiffer and Meyburg 2015). Measurements of nestlings with known hatching date (N = 49) were analysed in a linear mixed model (Bates et al. 2015) to compute an aging curve for red kite nestlings in the study area. We used the eighth primary feather length as response variable and age as explanatory variable. We further included hatching rank (first, second and third), food supplementation and sex as explanatory variables into the analysis. Brood ID and bird ID were included as random effects.

Growth curves often are of sigmoid nature. Due to only few measurements in the non-linear phase, we only analysed the linear phase. Therefore, 18 measurements that were taken before an age of 15 days were not included in the analysis, as they would have had a strong influence on the slope of the linear curve. This led to a sample size of 139 measurements.

At the same individual age, birds with different hatching rank showed differences in the length of their eighth primary: nestlings with hatching rank three had 17.02 mm (CrI = -24.67, -9.42) shorter feathers, and second-hatched 5.11 mm (CrI = -10.28, 0.09) shorter feathers than nestlings with hatching rank one. Food supplementation significantly influenced feather growth resulting in a length difference of 9.85 mm (CrI = 18.15, 0.67) between treated and control nestlings at the same age. Sex was excluded from the analysis as it showed no significant effect. The resulting equation was used to age 552 nestlings without known hatching date. Third-hatched nestlings sometimes showed very high negative residuals resulting in (1) considerably younger age estimates when using the equation for aging than nest observation suggest, and thus (2) large age differences between siblings. However, the highest difference in hatching date between consecutive siblings of known hatching date was four days. Therefore, we adjusted the calculated hatching date of the last-hatched nestling so that the difference to the next sibling was not more than four days.

For nestlings with no primary feather measurement we calculated a wing length growth curve, for which we used the same model structure as for the feather growth curve. Due to missing wing lengths and feather lengths six nestlings could not be aged.

# S2: Natural food availability

Small rodents, especially voles (*Microtus arvalis* and *Arvicola terrestris*), are an important food resource for red kites during breeding (Aebischer 2009; Wasmund 2013). We, therefore, used rodent activity as a proxy for natural food availability. Our rodent activity survey was based on the rodent transect method of Apolloni et al. (2018). We searched once a month for traces of rodents (runways, holes and heaps) on transects (length = 5 m, width = 1 m) in four sub-areas within the study area. In each sub-area, we selected nine transects per habitat type: meadow, winter cereal, other crops, forest edge and field edge structures.

A generalized linear model using the package lme4 (Bates et al. 2015) with negative binomial error distribution and a log-link function was used to analyse annual and seasonal variation in rodent activity. The number of traces found in a transect was used as response variable. All transects from the four sub-areas were analysed together (N = 665 transects). Habitat type, year and month of measurement were entered as explanatory variables. It was not always possible to find nine transects per habitat type due to differences in land use between sub-areas. Therefore, the log of the monthly number of available transects per habitat type was included as offset. As a proxy for the red kites’ natural food availability, we used the predicted monthly traces in meadows (denoted as rodent activity), because meadows represent the main vole-foraging habitat of red kites (Aebischer 2009; Pfeiffer and Meyburg 2015; Fig. S2).


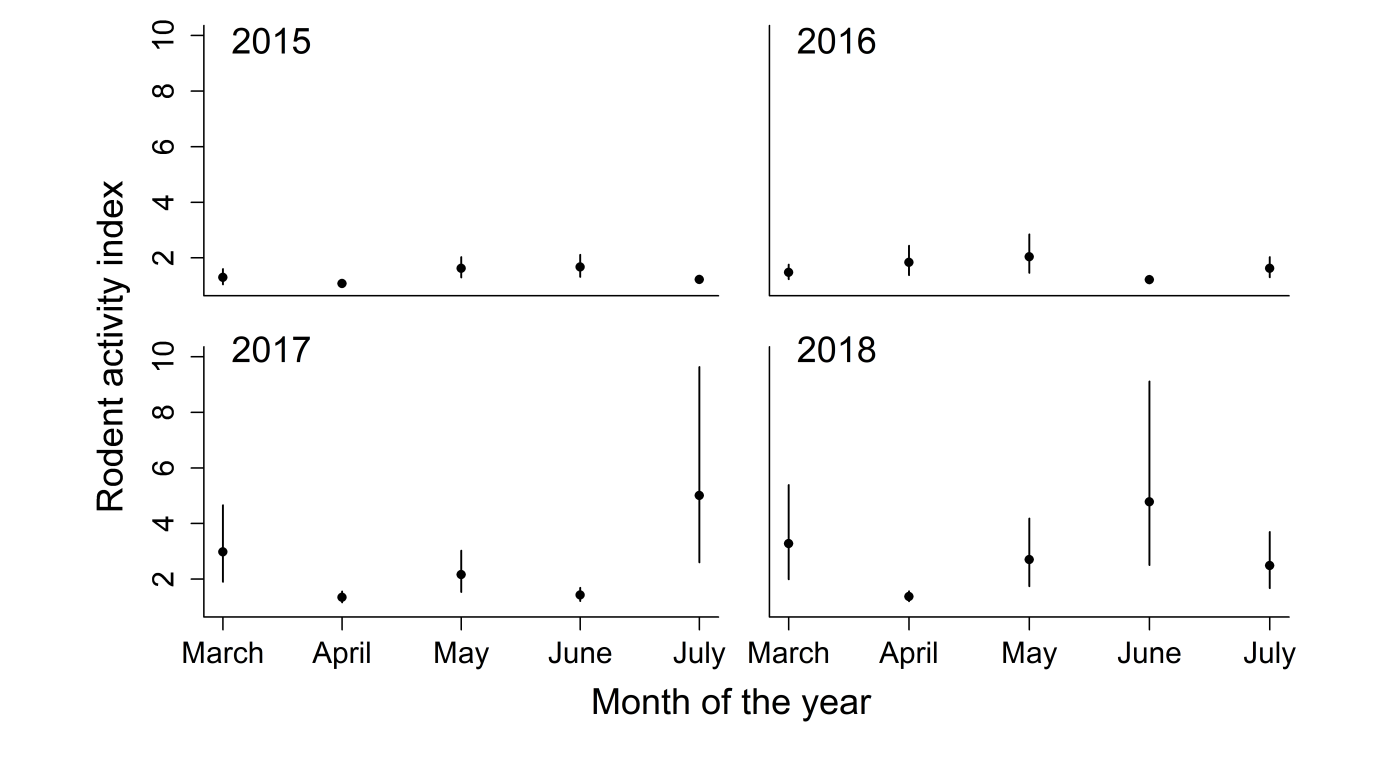
 **Fig. S2** Monthly rodent activity (in meadows) during the breeding period. Error bars represent standard errors

References

Aebischer A (2009) Der Rotmilan. Ein faszinierender Greifvogel, 1st edn. Haupt, Bern.

Apolloni N, Grüebler MU, Arlettaz R, Gottschalk TK, Naef-Daenzer B (2018) Habitat selection and range use of little owls in relation to habitat patterns at three spatial scales. Animal Conservation:1–11 doi: 10.1111/acv.12361.

Bates D, Mächler M, Bolker BM, Walker SC (2015) Fitting Linear Mixed-Effects Models Using lme4. Journal of Statistical Software 67:11–48 doi: 10.18637/jss.v067.i01.

Mougeot F, Garcia JT, Viñuela J (2011) Breeding biology, behaviour, diet and conservation of the red kite (Milvus milvus), with particular emphasis on Mediterranean populations Ecology and conservation of European dwelling forest raptors and owls, pp 190–204.

Pfeiffer T, Meyburg B-U (2015) GPS tracking of Red Kites (Milvus milvus) reveals fledgling number is negatively correlated with home range size. J Ornithol 156:963–975 doi: 10.1007/s10336-015-1230-5.

Traue H, Wuttky K (1966) Die Entwicklung des Rotmilans (Milvus milvus L.) vom Ei bis zum flüggen Vogel. Beiträge zur Vogelkunde 11:253–275.

Wasmund N (2013) Der Rotmilan (Milvus milvus) im Unteren Eichsfeld. Brutbestand, Nahrungsökologie und Gefährdungsursachen. Dissertation, Georg-August-Universität, Göttingen.
